# Determination of 14 Benzodiazepine Multiresidues in Aquaculture Environment by Ultra-High-Performance Liquid Chromatography–Tandem Mass Spectrometry

**DOI:** 10.3390/molecules30040775

**Published:** 2025-02-07

**Authors:** Hongyang Guo, Jianwu Chen, Guangjun Jiang, Yuqing Mei, Zhiqiang Gong, Mingdian Liu, Jinping Li, Jinhua Gan

**Affiliations:** 1 School of Environmental Engineering, Wuhan Textile University, Wuhan 430073, China; guohongyang1999@163.com; 2 Yangtze River Fisheries Research Institute, Chinese Academy of Fishery Sciences, Wuhan 430223, China; chjw@yfi.ac.cn (J.C.); g03152730@126.com (Y.M.); 18971373183@163.com (Z.G.); lovejingxuan@163.com (M.L.); 3 School of Agriculture, Ludong University, Yantai 264025, China; 15937160974@163.com

**Keywords:** ultra-high-performance liquid chromatography–tandem mass spectrometry, benzodiazepines, aquaculture environment, multiresidues

## Abstract

In this study, an analytical method for the simultaneous determination of 14 benzodiazepine (BDZ) multiresidues in aquaculture environmental water and sediment was developed using ultra-high-performance liquid chromatography–tandem mass spectrometry (UHPLC-MS/MS). The method uses an internal standard for quantification and achieves chromatographic separation and analysis within 11 min. The results of method validation showed that the recoveries of most analytes were in the range of 70–120% in water or sediment matrices, and the correlation coefficients of the 14 target chemistries were R^2^ > 0.99, with relative standard deviations (RSD) < 15%. The limits of detection (LODs) and the limits of quantification (LOQs) were in the ranges of 0.002–0.01 μg/L and 0.01–0.03 μg/L for water and 0.01–0.5 μg/kg and 0.04–1 μg/kg for the sediment matrix. The method is simple and has high rapidity, high sensitivity, and low cost. It provides technical support for the simultaneous monitoring of BDZ residues in the aquaculture environment.

## 1. Introduction

Benzodiazepines (BDZs) have recently been used as anesthetics in the sedation and hypnosis of aquatic animals as well as the transport and temporary sale of fresh aquatic products. As a result, BDZ residues are found in aquaculture environments such as water bodies and sediments, posing potential health hazards. BDZs are derivatives of 1,4-benzodiazepines containing two benzene rings and a seven-atom heterocycle, which bind to brain receptors. They act as sedatives, drugs, and anticonvulsants based on their effects on different regions of the brain [1]. Thus, they are used to treat anxiety, epilepsy, insomnia, and alcohol withdrawal syndromes and are the most commonly used sedative in clinical practice [2]. Long-term intake of BDZs can cause health hazards such as drowsiness, fatigue, mental disorders, reduction in white blood cells, and increased liver burden. These side effects are particularly serious in patients with cardiovascular disease and can cause addiction in long-term users [3,4,5,6]. BDZs are administered close to their toxic and lethal dosage, and their overdose can cause severe memory loss and death. BDZs also increase the severity and mortality of many diseases [7]. Their misuse or illicit use globally is evidenced by the increased concentrations of lorazepam, clonazepam, and temazepam in wastewater in the United States and Mexico [8].

Many BDZs are resistant to photodegradation, which increases their persistence in aquaculture water. Sediments adsorb nonpolar compounds and pollutants that are poorly soluble in the water; thus, BDZs will inevitably affect aquatic plants that depend on sediments for their growth [9,10]. BDZs accumulated in aquatic plants will enter the systems of aquatic animals via circulation and cause secondary pollution [11]. They can also accumulate in aquatic products and are potentially hazardous. At present, studies on the detection of BDZ residues focus mainly on matrices such as blood, hair, and urine [12,13,14], but there are few studies on BDZ residues in the aquaculture environment (water and sediment). In addition, most methods detect one or a few common BDZs. For example, Aitor et al. [15] used molecularly imprinted polymers combined with ultra-high-performance liquid chromatography–mass spectrometry (UHPLC-MS) to detect diazepam, and Mahsa et al. [16] used dual solvent stir bar microextraction (DSSBME) combined with high-performance liquid chromatography–ultraviolet detection (HPLC-UV) to detect lorazepam and clozapine. However, specific detection methods have not been proposed for many other less common BDZs. Thus, BDZ pollution in the aquaculture environment cannot be timely and accurately studied to determine their safety hazards and predict associated accidents. Therefore, detection methods for BDZs in water and sediment in the aquaculture environment must be developed to assess the associated risks.

Immunoassays [17,18], electrochemical analysis [19,20], Raman spectroscopy, gas chromatography–mass spectrometry (GC–MS) [21,22], high-performance liquid chromatography (HPLC) [23,24], and high-performance liquid chromatography–tandem mass spectrometry (HPLC–MS/MS) are the primary methods used for BDZ detection [25,26]. Immunoassays are usually performed on batch samples, whereas GC–MS generally requires derivatization and is cumbersome as well as time-consuming. High-performance liquid chromatography–ultraviolet detection (HPLC-UV) has low sensitivity and susceptibility to interference from impurities, and the simultaneous analysis of multicomponent compounds using this approach is time-consuming. In this study, the UHPLC-MS/MS method was optimized for detecting 14 BDZ residues in aquaculture environmental samples, expanding its application beyond traditional BDZ detection in biological samples. The method achieves chromatographic separation and detection in just 11 min, significantly improving analysis speed compared to traditional methods, which require more time. Additionally, it offers LODs of 0.002–0.01 μg/L (water) and 0.01–0.5 μg/kg (sediment) and LOQs of 0.01–0.03 μg/L (water) and 0.04–1 μg/kg (sediment), both much lower than existing methods, enabling higher sensitivity for detecting trace BDZ residues in the environment. Furthermore, this study simplifies sample pretreatment, reduces testing time and cost, and increases throughput, making it more suitable for large-scale environmental monitoring. This optimized method provides a more efficient, sensitive, and cost-effective solution for aquaculture pollution control and food safety supervision.

**Figure 1:**
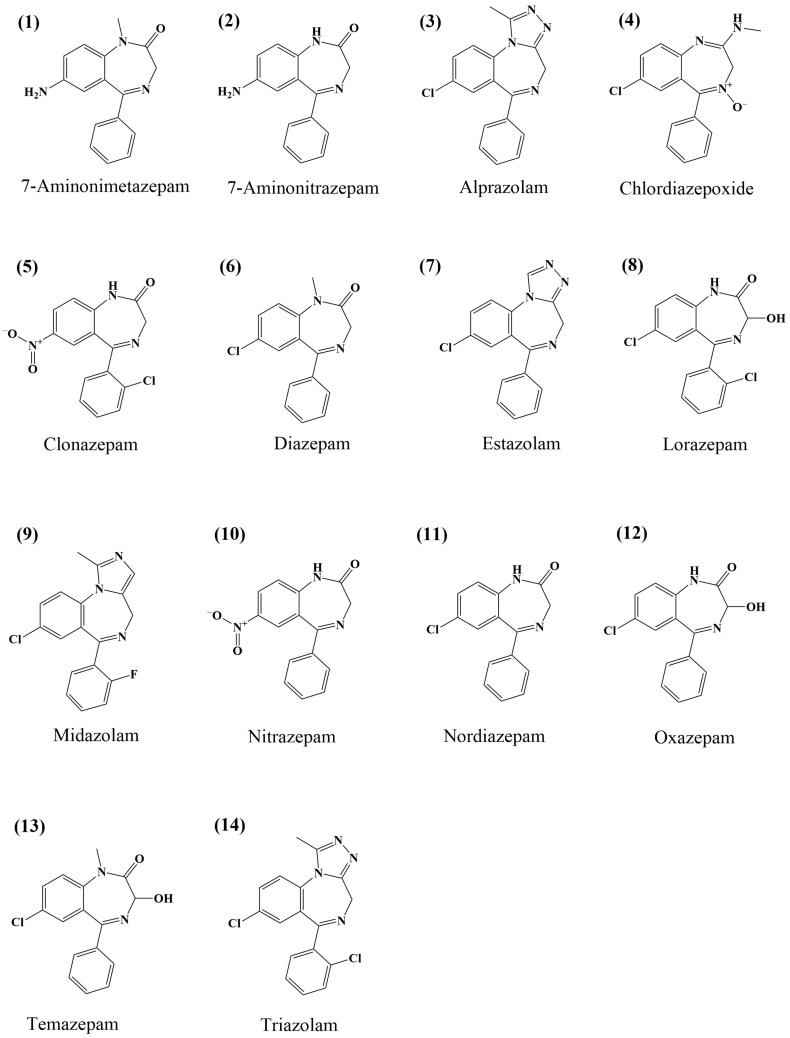
Structures of analyzed 14 benzodiazepines.

Herein, 14 BDZ residues (Figure 1) in aquaculture water and sediments were simultaneously determined using ultra-high-performance liquid chromatography–tandem mass spectrometry (UHPLC–MS/MS). In addition to its simplicity and efficiency, this method exhibits enhanced sensitivity and effective recovery. This makes it suitable for the rapid detection of samples. UHPLC–MS/MS can also provide technical support to the relevant regulatory authorities for monitoring BDZ residues in environmental sediments.

## 2. Materials and Methods

### 2.1. Instruments and Reagents

A liquid chromatography–mass spectrometer (Thermo Fisher, TSQ Quantiva, Waltham, MA, USA); an ultrasonic oscillator (Ultrasonic Instrument Co., Ltd., Kunshan, China); a nitrogen blower (ANPEL Hi-temp Dry Bath, Shanghai, China); a vortex mixer (North TZ-Biotech Develop. Co., Beijing, China); a centrifuge (BMH Ltd. sigma 3k15, Thermo Fisher, Waltham, MA, USA); a rotary evaporator (Buchi R-300, Beijing, China); and an electronic balance with an accuracy of 0.0001 g (MettlerToledo International Inc., Zurich, Switzerland) were used herein.

A total of 10 mixed labels were also used, such as diazepam (99.9% purity); nitrazepam (99.9% purity); clonazepam (99.9% purity); chlordiazepoxide (99.9% purity); oxazepam (99.9% purity); midazolam (99.4% purity); lorazepam (99.9% purity); estazolam (99.9% purity); alprazolam (99.9% purity); and triazolam (99.5% purity). The solvent for the standard product is methanol. The concentration of the standard product is 100 mg/L. The above standards were purchased from Alta Technology Co., Tianjin, China.

Five single standards, namely 7-aminonitrazepam (99.9% purity, 100 mg/L, acetonitrile); 7-aminometazepam (99.9% purity, 10 mg/L, acetonitrile); nordiazepam (99.9% purity, 100 mg/L, methanol); temazepam (99.9% purity, 100 mg/L, methanol); and D5-diazepam (99.9% purity, 100 mg/L, methanol), were used. These standards were purchased from Alta Technology Co., Tianjin, China.

Other materials included methanol, acetonitrile, n-hexane, and ethyl acetate (chromatographically pure, J.T. Baker Company, Phillipsburg, NJ, USA); ammonia solution (chromatographically pure, ≥25%, Aladdin, Shanghai, China); formic acid and acetic acid (chromatographically pure, Aladdin, Shanghai, China); anhydrous magnesium sulfate; NaCl (analytically pure, China National Pharmaceutical Group, Beijing, China); and experimental water (Watson’s drinking water).

### 2.2. Preparation of Stock, Working, and Calibration Curve

In this paragraph, we outline the preparation of stock solutions, working solutions, and calibration standards to establish a calibration curve for the analysis of benzodiazepines (BDZs) using an internal standard approach.

Preparation of the mixed standard stock solution: Take 10 mixed standard solutions and 4 single standard solutions, dilute with methanol to prepare a mixed standard stock solution containing 14 BDZs at 10 mg/L. Store at −18 °C with a validity period of 6 months.

Preparation of the mixed standard working solution: Pipette 100 μL of the mixed standard stock solution into a 10 mL volumetric flask, then dilute to volume with methanol to obtain a mixed standard working solution at 100 μg/L. Store at −18 °C and use within one week.

Preparation of the internal standard stock solution: Dilute the D5-diazepam standard solution with methanol to prepare an internal standard stock solution at 10 mg/L. Store at −18 °C with a validity period of 6 months.

Preparation of the internal standard working solution: Pipette 100 μL of the internal standard stock solution into a 10 mL volumetric flask, then dilute to volume with methanol to obtain an internal standard working solution at 100 μg/L. Store at −18 °C and use within one week.

Preparation of the calibration curve: Pipette 0.1, 0.2, 0.5, 1.0, and 2.0 mL of the mixed standard working solution (100 μg/L) into five separate 10 mL volumetric flasks. Add 0.5 mL of the internal standard working solution (100 μg/L) to each flask, then dilute to volume with methanol to obtain calibration standards with BDZ concentrations of 1, 2, 5, 10, and 20 μg/L, all containing an internal standard concentration of 5 μg/L.

By following these steps, a robust calibration curve can be established, allowing for precise and accurate analysis of BDZs in various samples.

### 2.3. Sample Pretreatment

#### 2.3.1. Sample Collection and Preparation

Water and sediment samples were collected from aquaculture environments in Hubei Province, China, according to the Chinese National Environmental Protection Standard (HJ 494-2009) [9]. The interference of other pollutants must be minimized during sampling. The same water sample was used as the blank control. This sample contains no target pollutants (BDZs) and serves primarily to correct for background interference and potential contaminants, while the water sample is used to analyze the concentration of BDZs. Aquaculture sediments were collected from the >6 cm-deep surface layer, and the sediment samples were freeze-dried for 72 h after removing twigs, large solid particles, and stones. A mortar and pestle was used to grind the seeds into powder, and a 100-mesh sieve was used to sieve them. After that, the powder was sealed in a sealed bag and kept at room temperature away from light.

#### 2.3.2. Extraction

Water samples: Aquaculture water was filtered through a glass fiber paper using a glass core filter device. A total of 100 mL of filtered water samples were accurately transferred to a 250 mL separatory funnel, and 50 μL of internal standard working solution as well as 20 mL of dichloromethane were introduced into it. Extraction was carried out by shaking, followed by adding 10 mL of 10% Na_2_CO_3_. The solution was mixed homogeneously and held for 10 min. The lower layer of the separatory funnel was placed in a pear-shaped bottle, and the residual liquid was repeated. In the separatory funnel, the residual liquid was subjected to the same method, and the extracts were combined. The resulting mixture was evaporated to dryness at 40 °C under reduced pressure. Then, 1 mL of 20% methanol in water (0.1% formic acid in water) was added to resolve the mixture, which passed through a 0.22 μm organic microporous filter membrane for UHPLC-MS.

Sediment samples: 2 g (accurate to 0.01 g) of the sample was weighed and placed in a 50-mL centrifuge tube. After adding 2 mL of purified water, 50 μL of the internal standard working solution with a concentration of 100 μg/L was added and thoroughly mixed with the sediment sample. The solution was held for 10 min, and 15 mL of 1% ammonia acetonitrile, 0.5 g NaCl, and 2 g anhydrous magnesium sulfate were added. The solution was subjected to vortex vibration for 2 min and ultrasonic extraction was performed for 5 min, followed by centrifugation for 5 min at 6000 r/min; this process was then repeated. The supernatant was extracted once and placed into a pear-shaped bottle; it was then dried via rotary evaporation at 40 °C. The pear-shaped bottle was placed in a nitrogen blower for 2 min to blow away ammonia. A total of 1 mL of 20% methanol–water (0.1% formic acid water) was added to resolve the solution accurately, which was analyzed on the machine using a 0.22 μm organic micropore filter membrane.

### 2.4. UHPLC–MS/MS Analysis

#### 2.4.1. Chromatographic Conditions

Column: Hypersil GOLD C18 HPLC (100 × 2.1 mm, 5 μm) liquid chromatography column; column temperature 40 °C; liquid phase flow rate of 0.30 mL/min. The mobile phases B and C were methanol and 0.1% formic acid in water. The injection volume was 10 µL, and the liquid chromatography conditions for gradient elution are shown in Table 1.

#### 2.4.2. Mass Spectrometry Conditions

The mass spectrometer model is the Thermo Scientific™ TSQ Quantis™ triple quadrupole mass spectrometer (Thermo Fisher, Waltham, MA, USA). Ion source parameters: H-ESI source, spray voltage: 3000 V(+), 2500 V(−); sheath gas: 40 arb; auxiliary gas: 10 arb; purge gas: 1 arb; ion transfer tube temperature: 350 °C; and vaporization temperature: 300 °C. A heated electrospray ion source was used to perform the parent ion scan in the positive ion mode, and an acquisition list of target compounds was created as the accessory data for target compounds that triggered secondary mass spectrometry. Table 2 shows the optimized mass spectrometry acquisition parameters.

### 2.5. Validation of Analytical Method

Analytical methods were validated as per EMA, FDA, and ICH guidelines [27,28]. The limits of detection and quantification (LODs and LOQs), calibration curves, and linearity, accuracy, precision, and matrix effect (ME) were assessed for the detection methods for 14 BDZs residues in water and sediment. In the analysis, the lowest concentration of the compound was used as a basis to calculate the LOD and LOQ by comparing signal-to-noise (S/N) ratios of 3 and 10 [29]. Measurement accuracy was evaluated by the recovery of each standard spike in blank samples at low, medium, and high levels. Precision was calculated for both intra-day and inter-day concentration levels as RSD (%) [30]. MEs were assessed based on the response values of blank matrix extracts spiked with the target in pure solvent. An internal standard was used for all calibrations and validation tests [31]. The robustness of the method was evaluated by testing its performance under small variations in experimental conditions. The method’s ability to maintain consistent results despite these changes was assessed by evaluating the impact of these variations on accuracy, precision, and matrix effects.

## 3. Results and Discussion

### 3.1. Optimization of Chromatographic Conditions

**Figure 2:**
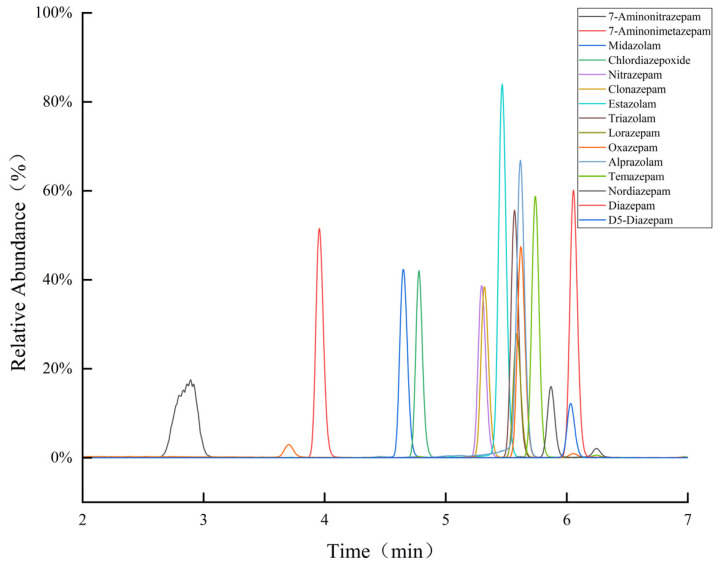
Extracted ion flow chromatogram of 14 benzodiazepines and 1 internal standard compound.

As moderately polar compounds, BDZs exhibit good retention properties in chromatographic analysis, particularly on C18 columns. Therefore, the Hypersil GOLD C18 HPLC (100 × 2.1 mm, 5 μm) liquid chromatography column was used to achieve effective separation and detection of BDZs as it has excellent separation efficiency and stability. The chemical structure of BDZs, typically derived from 1,4-BDZ, imparts specific characteristics that facilitate their reaction with acids and buffer salts in the mobile phase. The pH of the mobile phase can be effectively adjusted by adding appropriate amounts of acid and buffer salts to the mobile phase for optimizing the retention of target compounds on the column, the degree of ionization, sensitivity, and the formation of peaks [32], as well as improving the accuracy and reliability of the analysis. Results revealed that BDZs exhibited higher response values in the methanol–water system than in the acetonitrile–water system. This implied that the former system was more suitable for BDZ analysis. Hence, we opted for formic acid in water and methanol as the mobile phases, exploring the impacts of 0.1% and 1% formic acid aqueous solutions on the 14 target BDZ compounds. The target compounds showed higher response values, sharper peaks, and enhanced separation when 0.1% formic acid aqueous solution and methanol were used as the mobile phase. Thus, this combination was considered optimal for detecting the target compounds. The peak shape of 7-aminonitrazepam was poor because of its higher polarity. Figure 2 shows the extracted ion flow chromatograms obtained under this condition, which provided a reliable basis for subsequent drug analysis. In Figure 2, 7-Aminonitrazepam shows an imperfect Gaussian sharp peak at a retention time of 2.89 min, primarily due to the strong interaction between its high polarity and the polar stationary phase of the chromatographic column. This leads to a longer retention time and uneven elution, resulting in a broader and less symmetrical peak. On the other hand, an additional peak appears at the retention time of 6.03 min for Diazepam, with two more additional peaks appearing slightly later. An additional peak also appears before the retention time of 7-Aminonimetazepam (3.96 min). These peaks are likely the minor peaks of Oxazepam, Temazepam, and Nordiazepam. The retention times of these minor peaks differ significantly from that of the main peak, and their peak areas are relatively small, indicating that they may stem from impurities in the target sample, instrument noise, or solvent effects, and generally do not represent the presence of the target compound.

### 3.2. Optimization of Mass Spectrometry Parameters

BDZs contain secondary and tertiary amine groups that can easily combine with hydrogen ions to form positively charged quasi-molecular ions. The 14 BZDs, including diazepam, and an internal standard with a mass concentration of 1 mg/L were injected into the mass spectrometer via flow injection in the positive ion mode. Then, first-stage mass spectrometry in full scan mode was performed on target compounds in the *m*/*z* range of 200–400 to obtain the parent ion [M+H]^+^ of the target compounds. This ion was subjected to a secondary mass spectrometry scan using a product ion scan to determine the product ion information of the target. Two product ions with robust and consistent relative ion abundances were chosen to form qualitative and quantitative ion pairs with their respective parent ions [33]. The optimized mass spectrometry parameters are shown in Table 2.

### 3.3. Optimization of Water Sample Pretreatment Conditions

The extractants commonly used for BDZs are trichloromethane [34], dichloromethane, hexane [35], acetonitrile, and ethyl acetate [36]. The target matrices used are mostly hair, blood, and urine. BDZs are slightly soluble in water and readily soluble in organic solvents such as alcohols, acetonitrile, and ethyl acetate. Methanol, acetonitrile, and water are mutually soluble, making them unsuitable for extracting BDZ residues in water. Ethyl acetate and dichloromethane are commonly used for extracting BDZs in animal food matrices. Experimental investigations proved that the extraction effect of dichloromethane and ethyl acetate as extraction solvents for liquid–liquid extraction differ only slightly. However, dichloromethane can be easily separated from the aqueous specimen in the lower layer of the dispenser funnel and was therefore used as the extractant.

Water was not used in the C18, HLB, or MCX [37] solid-phase extraction columns for enrichment and concentration to ensure that BDZs can be effectively adsorbed. Moreover, the water samples contained small particles and organic matter, which could block the interpacking gap and increase the column enrichment operation time for a single batch of samples. Some water samples completely blocked the column although they were filtered through a glass fiber filter paper. Therefore, the liquid–liquid extraction method was used instead of solid-phase extraction.

### 3.4. Optimization of Sediment Pretreatment Conditions

BDZs are structurally similar; however, they have different solubility properties due to different substituents on the ring. BDZs are almost insoluble in water and soluble in organic solvents. Ethyl acetate and acetonitrile [38,39,40] are commonly used BDZ extraction solvents. Herein, acetonitrile was used as the extraction agent and its extraction efficiency was compared with those of ammonia acetonitrile and formic acid acetonitrile for the 14 BZDs.

**Figure 3:**
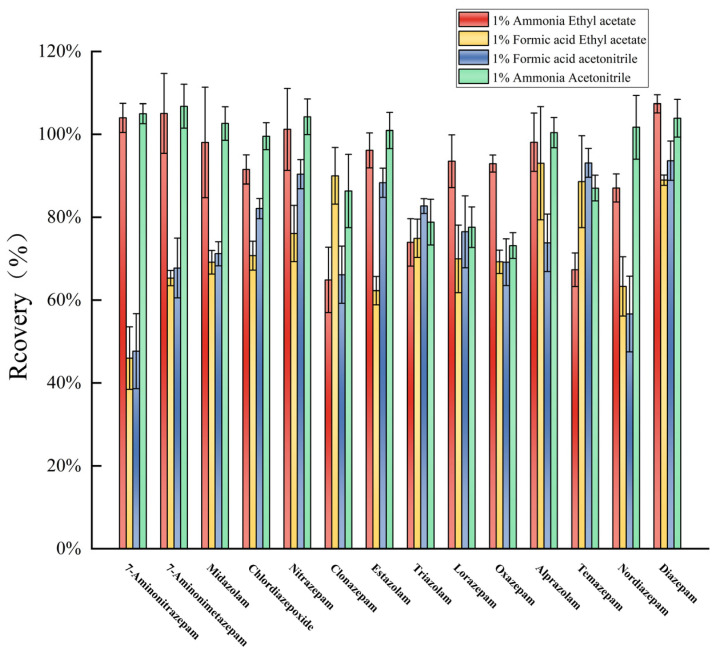
Extraction recoveries of different extractants.

**Figure 4:**
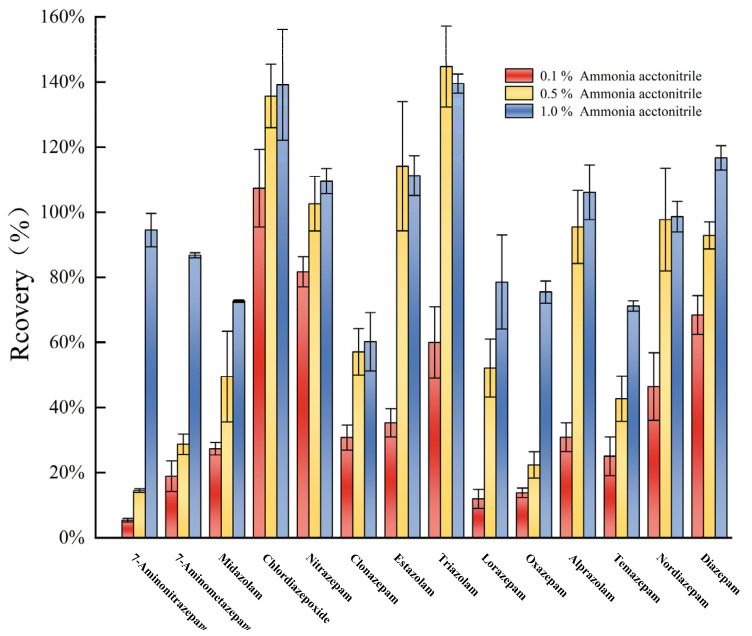
Extraction recoveries of different mass fractions (0.1 %, 0.5 %, and 1.0 %) of ammonia acetonitrile (n = 3).

Acetonitrile and ethyl acetate could not completely extract the 14 targets (Figure 3). Acetonitrile formic acid and ethyl acetate formic acid were less efficient, and ammoniacal acetonitrile and ammoniacal ethyl acetate were highly efficient. This suggests that the targets were mostly present in a molecular state under alkaline conditions [41] and could be easily extracted by organic solvents [2]. However, ammonia acetonitrile exhibited better extraction efficiency than ammonia ethyl acetate and was therefore used as the extractant herein. The extraction efficiencies of target analytes were also compared with different mass fractions of ammonia acetonitrile such as 0.1%, 0.5%, and 1.0% (Figure 4). The recoveries were mostly in the range of 70–120% when 1% ammonia acetonitrile was used as the extractant, and the extraction efficiencies were better than those of 0.1% and 0.5% ammonia acetonitrile. As shown in Figure 3 and Figure 4, the extraction efficiency of some target compounds is relatively high, even approaching 120% or 140%, mainly due to matrix effects. However, since the focus of this stage of the study was on the selection of extractants, matrix effects were not fully considered at this stage. This indicates that matrix effects caused the solvent calibration curve to inaccurately reflect the true concentration of the target compounds in the samples. Therefore, to correct for matrix effects, a blank matrix-matched calibration curve will be used in the following studies.

Brine was added during sample pretreatment to stratify the extraction solvent and the aqueous phase. Moreover, it prevented water and water-soluble polar matrix interferences in the sample from entering the extraction solution, thus reducing interference with the target and preventing the contamination of the mass spectrometry ion source [38,42]. Anhydrous magnesium sulfate was added to effectively remove the water in the extract and obtain an accurate volume of sample solution after concentration and resolubilization, thus improving the target recovery. Brine and anhydrous magnesium sulfate were used as the salting-out agent and water remover, respectively, and their dosages were optimized. Experimental results showed that when 0.5 g of brine and 2 g of anhydrous sodium sulfate were added, the sample extract had the best salting-out effect and high target extraction efficiency was achieved.

### 3.5. Method Validation

#### 3.5.1. Linear Range and Sensitivity

The blank matrix standard curve is plotted using the ratio of the peak area of the measured component and the internal standard peak area y and the mass concentration x as the vertical and horizontal coordinates, respectively. The LOD and LOQ of each target were determined based on S/N ratios of 3 and 10 [43,44], respectively. The linear ranges, matrix standard curves, R^2^, LODs, and LOQs of the 14 BDZs in the sediment are shown in Table 3 and Table 4.

As shown in Table 3, the R^2^ of the 14 BDZs in the water samples was >0.99 in the appropriate linear ranges. The optimized pretreatment process for water samples showed good extraction results for the 14 BDZs. The LOD and LOQ ranges of the validated method were 0.002–0.010 μg/L and 0.010–0.030 μg/L, respectively.

As shown in Table 4, the R^2^ of the 14 BDZs in the sediment samples was >0.99 in the appropriate linear ranges. The optimized pretreatment process for sediment samples showed good extraction efficiencies for the 14 BDZs. The LOD and LOQ ranges of the validated method were 0.01–0.50 μg/kg and 0.04 μg/kg–1.00 μg/kg, respectively.

#### 3.5.2. Matrix Effect

When target compounds are detected by UHPLC–MS/MS, components other than the measured substance in the sample considerably interfere with the analysis and affect the accuracy of the results. Such interferences and influences are known as the ME, which manifest as ion suppression [45] or ion enhancement. ME adversely affects linearity, quantification, detection, accuracy, and precision [46,47]. Interfering substances that produce MEs are classified into two categories: (1) Endogenous substances that originate from the analyte itself and are retained in the final extract, including salts, strong polar compounds, surfactants, and lipids, amines, and peptides that are structurally similar to the target compounds. (2) Exogenous substances that are not derived from the matrix itself but from the external environment during the method setup, including plastic and polymer residues, ion-pairing reagents, organic acids, and buffer solutions. Therefore, MEs should be evaluated when setting up UHPLC–MS/MS, and appropriate methods should be used to reduce or compensate for the ME to ensure the accuracy and reliability of the detected data.

The ME is commonly evaluated via postcolumn injection [48] and postextraction addition. Postcolumn injection uses liquid chromatography to feed a blank sample extract, and a certain concentration of target compounds is propelled into the mass spectrum using a three-way mixed-liquid-phase postcolumn effluent and a needle pump. Mass spectrometry is used to collect a map for exploring the region of ionization inhibition or enhancement when interfering substances elute from the column. ME can also be observed in real time for each analysis time period; however, it cannot be quantified. The postextraction addition method can be used for the quantitative assessment of MEs. It has been mentioned in various regulatory documents for evaluating the ME [45]. The ME is typically determined by comparing the sample response after extraction and addition of the substance to be measured with the response value of the substance in a pure solution [49,50,51], which is calculated as:

ME (%) = (Area B/Area A) × 100%

Area B represents the peak area of the target compound after extraction. Pure solution peak area is designated as Area A. In cases wherein the tested samples were derived from different individual matrices and an inhomogeneous ME caused an error in the detection result, same concentration of compounds were tested using the analogs or isotopes of compounds as the internal standard. The ME calibrated using the internal standard was introduced here, and a range of 0.8–1.2 is considered acceptable. The calculated coefficient of variation of the internal standard–calibrated ME must be <15%. When the ME is more serious, isotope-labeled internal standard is recommended. This is because it can maximally counteract the ME during the extraction and ionization of samples because of their similar chemical nature [51]. This can be calculated as follows:

IS-corrected ME (%) = (Area ratio B/Area ratio A) × 100%

After extraction, the area ratio B compares the target and internal standard response values. In a pure solution, the area ratio A compares the target and internal standard response values. The ME manifests as a value at 100%, and a value below and above 100% signifies signal suppression (ionization suppression) and signal enhancement (ionization enhancement), respectively [50]. Herein, the MEs of 14 BDZs in water and sediment samples were compared at three different levels of addition: low, medium, and high.

Table 5 shows the MEs in water samples for the 14 target compounds analyzed here. The spiked levels ranged from low to high between −20% and 20% for all 14 target compounds, with lower MEs indicating that the internal standard can correct the MEs better.

Table 6 reports the ME of the 14 target compounds in the sediment samples. For lorazepam, the ME ranged from low to high as the ionization enhancement ranged from 26.3% to 50.3%. For alprazolam, the MEs ranged from low to high as the ionization enhancement and inhibition ranged from −17.4% to 63.3%. For the remaining 12 target compounds with low MEs, the surface internal standard could correct the MEs well.

#### 3.5.3. Accuracy and Precision

The accuracy and precision of the method were evaluated using the standard addition method, and the corresponding results are shown in Table 7 and Table 8.

The recovery and stability of the analyzed water samples showed that the recoveries of seven target compounds were higher than 120% when the spiked concentrations were 0.5 and 1 μg/L (Table 7); these were mainly compounds with strong polarity. The recoveries of the 14 target compounds were 70–120% when the spiked concentrations were 5 μg/L, which proved the enhanced accuracy of the screening method. When the spiked concentration was 5 μg/L, the recoveries of the 14 target compounds were between 70% and 120%; this proved the enhanced accuracy of the screening method. During stability evaluation, the RSDs of target compounds were <15% at three different spiked levels. The results showed that the proposed method has good reproducibility and high accuracy for the determination of BDZ residues in water. This is important for monitoring BDZ residues in the aquaculture environment.

Table 8 shows that the recoveries of 7-aminonitrazepam, lorazepam, oxazepam, alprazolam, and temazepam at low concentrations were >120%, and those of most of the other target compounds were between 70% and 120% at the low concentrations, and those of the 14 target compounds at middle and high concentrations were 77.1–99.7% in the sediment samples. The recoveries of the 14 target compounds in the middle and high concentrations of the sediment samples ranged from 77.1% to 99.7%, which were in line with the requirements of multicomponent detection. Thus, the proposed method is suitable for routine analysis with high recovery and good precision and can be used for the simultaneous determination of 14 BDZs in real bottom mud samples.

### 3.6. Comparison with Other Methods

As shown in Table 9 the following is a comparison of the different analytical methods based on matrix, linear range and detection limit.

## 4. Conclusions

In this study, the extraction methods for 14 BDZs in water and sediment were optimized and validated by examining parameters such as linearity, LOD, LOQ, ME, precision, and accuracy.

1. The recoveries of most BDZs ranged from 70% to 120% at three different concentration levels for both water and sediment matrices, and the intra- and inter-day RSDs were <15% at the three different concentration levels. The LOD and LOQ ranges were 0.002–0.01 μg/L and 0.01–0.03 μg/L and 0.01–0.50 μg/kg and 0.04–1.00 μg/kg for the water samples and sediment matrix, respectively.
2. The proposed method is highly sensitive and has good recovery. It is simple, efficient, rapid, and low-cost and could simultaneously analyze and detect 14 BDZs.
3. With potential applications such as locating contamination hotspots, monitoring compliance with permissible limits, and evaluating how various technologies reduce the presence of these contaminants, this method provides technical support to detect BDZ residues in aquaculture environments.

## Figures and Tables

**Table 1:** Liquid chromatography gradient elution procedure.

Time (min)	B (%)	C (%)
0–0.5	10	90
0.5–2.0	40	60
2.0–2.5	60	40
2.5–8.0	90	10
8.0–9.1	10	90
9.1–11	10	90

**Table 2:** Parameters for mass spectrometry analysis of benzodiazepines.

Compound	Precursor Ion (*m*/*z*)	Product Ion (*m*/*z*)	Collision Energy (V)	Retention Time (min)
7-Aminonitrazepam	252.2	121.1 *	25.35	2.89
		224.1	19.49	
7-Aminonimetazepam	266.2	135.1 *	24.17	3.96
		209.1	23.49	
Midazolam	326.2	291.1 *	24.55	4.65
		249.1	34.19	
Chlordiazepoxide	300.2	227.1 *	23.37	4.78
		283.1	12.16	
Nitrazepam	282.2	236.1 *	22.61	5.29
		180.1	34.99	
Clonazepam	316.1	270.0 *	23.41	5.32
		214.1	35.92	
Estazolam	295.2	267.1 *	22.57	5.47
		205.1	38.36	
Triazolam	343.1	308.1 *	25.05	5.57
		239.0	38.70	
Lorazepam	321.1	275.1 *	20.37	5.59
		229.1	28.59	
Oxazepam	287.2	241.1 *	20.80	5.62
		269.0	13.21	
Alprazolam	309.1	281.0 *	24.92	5.63
		205.1	39.92	
Temazepam	301.1	255.0 *	20.92	5.74
		177.1	37.10	
Nordiazepam	271.1	140.1 *	25.98	5.87
		165.1	26.65	
Diazepam	285.2	193.1 *	30.06	6.06
		154.1	25.05	
D5-Diazepam	290.2	198.2 *	31.33	6.03

* quantitative ion.

**Table 3:** Matrix standard curves, correlation coefficients, limits of detection, and limits of quantification of 14 benzodiazepines in water samples.

Compound	Linear Range (μg/L)	Standard Curve	R^2^	LOD (μg/L)	LOQ (μg/L)
7-Aminonitrazepam	1~20	y = 0.6632x − 0.2578	0.9939	0.010	0.030
7-Aminometazepam	1~20	y = 0.6396x − 0.1917	0.9910	0.008	0.030
Midazolam	1~20	y = 0.7182x − 0.1904	0.9968	0.004	0.010
Chlordiazepoxide	1~20	y = 0.6944x − 0.1643	0.9934	0.004	0.010
Nitrazepam	1~20	y = 0.7837x − 0.2544	0.9924	0.005	0.020
Clonazepam	1~20	y = 0.6022x − 0.1123	0.9928	0.003	0.010
Estazolam	1~20	y = 1.2087x − 0.2424	0.9959	0.002	0.010
Triazolam	1~20	y = 0.6004x − 0.1367	0.9959	0.003	0.010
Lorazepam	1~20	y = 0.7173x − 0.2568	0.9919	0.006	0.020
Oxazepam	1~20	y = 1.0835x − 0.3744	0.9927	0.005	0.020
Alprazolam	1~20	y = 0.7672x − 0.2215	0.9945	0.003	0.010
Temazepam	1~20	y = 1.8753x + 0.621	0.9971	0.004	0.010
Nordiazepam	1~20	y = 0.2429x + 0.0558	0.9965	0.004	0.010
Diazepam	1~20	y = 0.8432x − 0.0293	0.9991	0.004	0.010

**Table 4:** Matrix standard curves, correlation coefficients, limits of detection, and limits of quantification for 14 benzodiazepines in sediment samples.

Compound	Linear Range (μg/L)	Standard Curve	R^2^	LOD (μg/kg)	LOQ (μg/kg)
7-Aminonitrazepam	1~20	y = 0.5021x − 0.3507	0.9971	0.50	1.00
7-Aminometazepam	1~20	y = 0.4486x − 0.4404	0.9908	0.03	0.10
Midazolam	1~20	y = 0.5562x − 0.8013	0.9946	0.02	0.05
Chlordiazepoxide	1~20	y = 0.3724x − 0.1371	0.9974	0.03	0.10
Nitrazepam	1~20	y = 0.4965x − 0.3090	0.9955	0.03	0.08
Clonazepam	1~20	y = 0.5298x − 0.0741	0.9992	0.50	1.00
Estazolam	1~20	y = 0.865x − 0.3691	0.9973	0.02	0.05
Triazolam	1~20	y = 0.6614x − 0.3945	0.9949	0.02	0.06
Lorazepam	1~20	y = 0.0664x − 0.0305	0.9949	0.20	0.70
Oxazepam	1~20	y = 0.1094x − 0.0619	0.9934	0.10	0.40
Alprazolam	1~20	y = 0.9659x − 0.6602	0.9917	0.01	0.04
Temazepam	1~20	y = 1.1089x − 0.5865	0.9929	0.02	0.07
Nordiazepam	1~20	y = 0.3578x − 0.2268	0.9915	0.04	0.10
Diazepam	1~20	y = 0.3075x − 0.099	0.9990	0.03	0.10

**Table 5:** Matrix effects in water samples at different quality control levels (n = 3).

Compound	Spiked (μg/L)	Matrix Effect (%)	RSD (%)
7-Aminonitrazepam	0.5	108.33	12.02
	1.0	84.56	8.80
	5.0	109.57	3.18
7-Aminometazepam	0.5	111.45	14.13
	1.0	102.60	3.94
	5.0	105.48	6.69
Midazolam	0.5	118.26	4.46
	1.0	114.77	5.73
	5.0	107.28	1.50
Chlordiazepoxide	0.5	119.57	11.10
	1.0	114.77	5.96
	5.0	87.10	8.23
Nitrazepam	0.5	92.59	4.47
	1.0	95.72	1.72
	5.0	97.68	5.18
Clonazepam	0.5	117.53	8.63
	1.0	91.01	2.43
	5.0	95.53	14.25
Estazolam	0.5	108.16	7.49
	1.0	84.55	6.08
	5.0	90.90	7.08
Triazolam	0.5	119.09	2.24
	1.0	117.70	3.07
	5.0	91.84	5.65
Lorazepam	0.5	118.92	1.58
	1.0	117.37	10.34
	5.0	119.63	9.92
Oxazepam	0.5	119.61	5.20
	1.0	115.15	3.99
	5.0	93.38	9.06
Alprazolam	0.5	119.62	6.43
	1.0	98.36	10.22
	5.0	92.70	6.18
Temazepam	0.5	118.15	4.66
	1.0	115.31	13.5
	5.0	88.03	5.47
Nordiazepam	0.5	81.39	7.68
	1.0	103.76	14.36
	5.0	106.11	4.60
Diazepam	0.5	118.07	6.72
	1.0	94.49	14.19
	5.0	98.16	3.26

**Table 6:** Matrix effects in sediment samples at different quality control levels (n = 3).

Compound	Spiked (μg/kg)	Matrix Effect (%)	RSD (%)
7-Aminonitrazepam	0.5	84.56	7.29
	2.5	108.33	11.4
	5.0	87.10	4.64
7-Aminometazepam	0.5	80.15	5.67
	2.5	95.72	8.81
	5.0	97.68	9.19
Midazolam	0.5	118.26	6.19
	2.5	115.31	3.56
	5.0	101.49	10.42
Chlordiazepoxide	0.5	114.77	9.55
	2.5	103.76	5.3
	5.0	96.11	6.4
Nitrazepam	0.5	91.84	10.97
	2.5	98.16	7.99
	5.0	94.49	3.32
Clonazepam	0.5	88.03	2.5
	2.5	81.20	12.59
	5.0	91.01	3.21
Estazolam	0.5	80.06	9.31
	2.5	83.55	3.3
	5.0	90.90	3.25
Triazolam	0.5	118.07	14.68
	2.5	120.09	12.37
	5.0	107.70	2.01
Lorazepam	0.5	150.25	8.66
	2.5	130.70	10.76
	5.0	126.30	7.51
Oxazepam	0.5	93.38	11.48
	2.5	115.15	4.65
	5.0	121.28	3.87
Alprazolam	0.5	163.29	11.47
	2.5	138.77	14.7
	5.0	82.56	2.16
Temazepam	0.5	95.92	11.49
	2.5	102.60	6.74
	5.0	109.57	13.8
Nordiazepam	0.5	117.53	7.25
	2.5	90.61	8.03
	5.0	111.45	9.63
Diazepam	0.5	108.16	3.55
	2.5	118.90	11.19
	5.0	118.11	10.78

**Table 7:** Accuracy and precision of 14 benzodiazepines in water samples (n = 6).

Compound	Spiked (μg/L)	Precision (Intra-Day)		Precision (Inter-Day)	
		Recovery (%)	RSD (%)	Recovery (%)	RSD (%)
7-Aminonitrazepam	0.5	132.0	5.5	122.4	4.9
	1.0	116.2	4.1	108.5	5.4
	5.0	99.3	8.2	97.8	6.2
7-Aminometazepam	0.5	124.7	4.8	119.2	4.3
	1.0	118.5	3.3	110.5	3.8
	5.0	110.7	5.2	104.7	6.9
Midazolam	0.5	113.7	5.3	108.8	7.3
	1.0	113.5	8.8	106.7	5.3
	5.0	103.7	4.2	109.8	4.2
Chlordiazepoxide	0.5	115.7	2.7	112.7	3.1
	1.0	114.3	5.8	111.2	4.6
	5.0	107.2	5.1	103.9	8.9
Nitrazepam	0.5	146.7	4.7	137.5	4.9
	1.0	119.3	4.8	113.4	5.7
	5.0	94.0	6.0	102.3	6.8
Clonazepam	0.5	123.7	5.8	125.8	7.3
	1.0	112.2	5.3	108.7	3.1
	5.0	104.5	3.3	106.9	4.6
Estazolam	0.5	112.8	2.6	109.9	3.7
	1.0	118.9	5.1	115.8	3.4
	5.0	101.6	7.0	111.9	5.9
Triazolam	0.5	113.1	3.2	107.7	8.1
	1.0	114.4	2.3	115.8	3.2
	5.0	102.2	9.5	104.9	4.3
Lorazepam	0.5	116.0	5.6	117.1	6.7
	1.0	113.4	4.2	109.3	4.5
	5.0	103.0	13.6	107.6	5.2
Oxazepam	0.5	119.8	6.8	111.9	5.9
	1.0	114.5	3.5	115.4	6.9
	5.0	95.8	4.2	99.7	3.2
Alprazolam	0.5	121.8	4.9	117.9	4.5
	1.0	117.9	3.1	109.7	3.6
	5.0	101.9	3.2	107.3	4.9
Temazepam	0.5	117.1	7.1	115.9	3.2
	1.0	114.1	5.3	108.7	7.4
	5.0	95.7	3.0	98.5	6.1
Nordiazepam	0.5	128.6	11.0	122.9	6.3
	1.0	116.7	6.8	117.8	4.5
	5.0	94.9	3.8	98.4	6.8
Diazepam	0.5	90.3	6.7	103.8	7.2
	1.0	114.0	4.4	109.4	6.4
	5.0	96.2	6.5	101.5	5.3

**Table 8:** Accuracy and precision of 14 benzodiazepines in sediment samples (n = 6).

Compound	Spiked (μg/L)	Precision (Intra-Day)		Precision (Inter-Day)	
		Recovery (%)	RSD (%)	Recovery (%)	RSD (%)
7-Aminonitrazepam	0.5	131.9	3.1	127.9	4.6
	1.0	94.0	6.2	98.2	5.1
	5.0	93.7	9.1	89.3	7.9
7-Aminometazepam	0.5	117.7	2.2	107.4	3.9
	1.0	99.7	9.2	97.3	4.7
	5.0	98.1	5.4	101.8	6.4
Midazolam	0.5	113.9	2.4	109.9	4.5
	1.0	95.3	3.7	105.8	3.3
	5.0	92.8	9.0	95.7	8.2
Chlordiazepoxide	0.5	112.2	3.1	115.9	4.2
	1.0	89.9	10.3	93.2	7.6
	5.0	82.5	11.0	88.7	3.4
Nitrazepam	0.5	109.8	5.1	113.2	3.2
	1.0	93.4	3.7	103.7	4.5
	5.0	77.7	7.7	94.4	6.6
Clonazepam	0.5	102.2	6.0	104.8	5.7
	1.0	86.8	4.7	96.7	5.2
	5.0	82.5	6.0	92.2	6.7
Estazolam	0.5	116.8	0.7	113.6	4.5
	1.0	84.2	5.0	89.7	4.9
	5.0	84.7	7.0	93.5	5.7
Triazolam	0.5	115.6	6.3	109.7	5.4
	1.0	95.5	5.3	103.5	6.1
	5.0	91.0	7.7	96.8	4.7
Lorazepam	0.5	130.5	4.1	133.7	6.4
	1.0	77.1	3.8	89.4	3.2
	5.0	80.1	10.5	91.1	2.7
Oxazepam	0.5	134.7	3.3	129.5	4.2
	1.0	76.6	8.2	83.3	5.9
	5.0	82.2	9.6	84.9	6.8
Alprazolam	0.5	148.0	4.4	131.1	7.1
	1.0	94.0	5.6	99.4	2.4
	5.0	88.6	7.7	92.3	4.5
Temazepam	0.5	126.1	3.7	118.9	5.6
	1.0	84.2	6.4	88.9	8.3
	5.0	77.9	6.4	87.8	4.1
Nordiazepam	0.5	117.3	2.5	113.7	5.2
	1.0	90.9	5.9	104.5	7.7
	5.0	86.0	12.2	98.0	4.3
Diazepam	0.5	114.2	3.2	117.2	7.2
	1.0	96.4	6.7	105.6	3.5
	5.0	90.7	8.7	103.3	6.2

**Table 9:** Comparison of present method with other reported methods.

Method	Matrix	Linear Range (μg/L)	LOD (μg/L)	Ref.
VAUS-ME-SFO/LC-MS/MS	Water, alcoholic, and non-alcoholic	0.124–7.810	0.316–0.968	[52]
AALLME–HPLC–UV	Water, plasma, and urine	2.3–800	0.7–0.9	[53]
GC-MS	Urine and blood	0.2–100	0.06–1.5	[14]
HPLC-DAD	Human plasma	3–500	1.26–2.09	[34]
UHPLC-MS/MS	Water and sediment	1–20	0.002–0.01 μg/L (water) 0.01–0.5 μg/kg (sediment)	This work

## Data Availability

The original contributions presented in this study are included in the article. Further inquiries can be directed to the corresponding author(s).
